# Superior mediastinal syndrome in paediatric B-cell acute lymphoblastic leukaemia: a case report

**DOI:** 10.3332/ecancer.2022.1457

**Published:** 2022-10-31

**Authors:** Rahat-Ul-Ain, Mahwish Faizan, Saba Fatima, Khalid Mehmood

**Affiliations:** 1 Department of Pediatric Hematology Oncology, University of Child Health Sciences, The Children’s Hospital Lahore, Lahore, Punjab 54000, Pakistan; 2 Department of Pediatric Radiology, University of Child Health Sciences, The Children’s Hospital Lahore, Lahore, Punjab 54000, Pakistan; 3 Department of Pathology, University of Child Health Sciences, The Children’s Hospital Lahore, Lahore, Punjab 54000, Pakistan

**Keywords:** superior mediastinal syndrome, anterior mediastinal mass, paediatric B-cell acute lymphoblastic leukaemia

## Abstract

Superior mediastinal syndrome secondary to an anterior mediastinal mass can be seen in acute lymphoblastic leukaemia (ALL) of T-cell lineage. We report a 3-year-old child with B-cell ALL, who presented with the superior mediastinal syndrome. The CT scan chest showed a huge anterior mediastinal mass and the peripheral blood immunophenotyping showed B-cell ALL. High-risk remission induction chemotherapy was given and he achieved remission by the end of induction therapy, both in terms of medullary and extramedullary disease, and is on maintenance chemotherapy now. This is the first reported case of a paediatric B-cell ALL presenting with superior mediastinal syndrome secondary to an anterior mediastinal mass.

## Introduction

B-cell acute lymphoblastic leukaemia (ALL), the most common paediatric malignancy, is generally never considered in the differential diagnosis of a paediatric malignant mediastinal mass. B-cell ALL presenting with an anterior mediastinal mass is extremely rare. Only two cases were reported in the literature [1, 2] with none of the cases seen in children so far. Primary malignant anterior mediastinal masses, in children, are not very common and most often are associated with Hodgkin lymphoma and T-lineage lymphoblastic lymphoma (LBL)/lymphoblastic leukaemia [3]. We report an unusual case of paediatric B-cell ALL presenting with the superior mediastinal syndrome secondary to an anterior mediastinal mass.

## Case report

**Figure 1.**
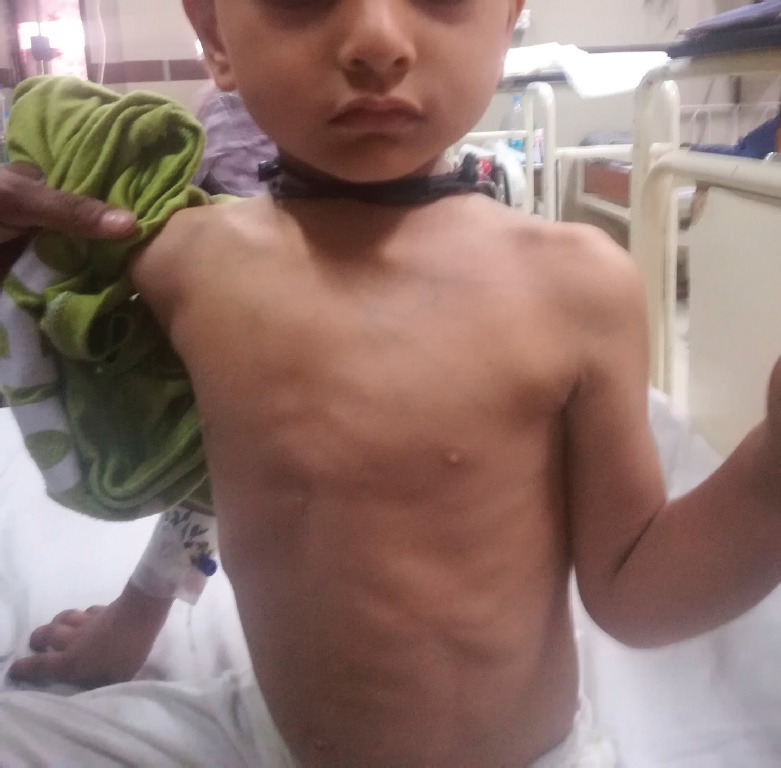
Distended veins over the chest along with subcostal and intercostal recessions.

**Figure 2.**
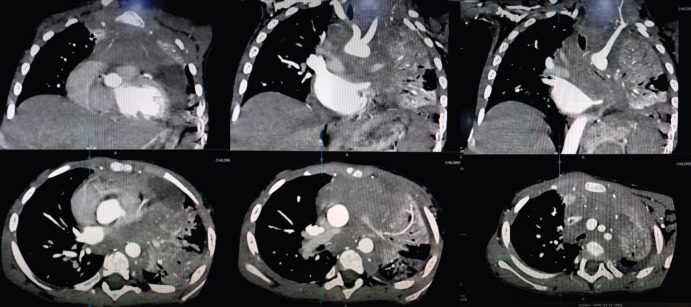
CT scan chest: A soft-tissue density lesion involving the anterior mediastinum extending down to left para-cardiac location. It collectively measures 41 × 50 ×60 mm (anteroposterior × craniocaudal × transverse) and causing compression of the left main stem bronchus and collapse consolidation of almost the whole of the left lung.

A 3-year-old boy presented via emergency in April 2021 with fever and progressive difficulty in breathing for 3 weeks, and difficulty lying down for 2 days. On examination, he was a thin, lean child, sitting in a tripod position with severe respiratory distress in the form of orthopnoea, nasal flaring and supra-sternal, subcostal and intercostal recessions. There were visible dilated veins over the upper part of the chest but there was no peri-orbital puffiness or upper limb oedema (Figure 1). The trachea was shifted towards the right side and there was a marked decrease in breath sounds on the left side of the chest. His chest X-ray was done in the emergency room, which showed opaque left hemithorax with left-sided pleural effusion and consolidation. Chest ultrasound confirmed mild-moderate pleural effusion with underlying lung consolidation and the suspicion of a mediastinal mass. His baseline complete blood counts showed marked leukocytosis (53.6 × 10^3^/uL), anaemia (7.6 g/dL), thrombocytopenia (20 × 10^3^/uL) and 15% lymphoblasts on peripheral blood film. There was no evidence of laboratory or clinical tumour lysis syndrome (TLS). A chest CT scan and flow cytometry on peripheral blood were done and high-dose steroids (methylprednisolone) were started for the management of the superior mediastinal syndrome. The CT chest confirmed an anterior mediastinal mass (Figure 2) and the flow cytometry showed a mononuclear cell population (blasts) in peripheral blood with the prominent expression of cluster of differentiation 10 (CD10), CD19, CD79a, Anti Terminal deoxynucleotidyl transferase (Tdt), Human Leukocyte Antigen - DR isotype (HLA-DR), Anti Kappa and Anti Lambda, suggestive of precursor B ALL. Cytogenetics was not done due to unaffordability.

The patient was given high-risk induction chemotherapy (UKALL 2019 guidelines). Orthopnoea started improving within 24 hours of steroid therapy and completely settled after 2 weeks. He also developed TLS and febrile neutropenia during induction chemotherapy. His day 29 minimal residual disease (MRD) showed less than 0.01% blasts and the post-induction CT scan showed complete resolution of anterior mediastinal mass with incidental finding of left eventration of the diaphragm. The patient successfully completed further cycles of chemotherapy with three episodes of febrile neutropenia. After 2 months of maintenance therapy, he was admitted with respiratory distress. It was suspected that he has developed bronchopneumonia but the chest CT-scan confirmed relapsed disease at the primary site of involvement (anterior mediastinal mass). He was given supportive care but the condition rapidly deteriorated and the patient expired.

## Discussion

This is the first reported case in a child with B-cell ALL presenting with superior mediastinal syndrome secondary to an anterior mediastinal mass. The index case is distinctive from the previous two reported cases (Table 1) as it presented in the early childhood period, not with the cardinal features of acute leukaemia but with the superior mediastinal syndrome with peripheral blood lymphoblasts. A few case series of acute leukaemia (both adult and paediatric cases) have reported the presence of mediastinal nodal involvement in a small percentage (2%–11%) of B-cell ALL, but none presented with the clinical features of a mediastinal mass [4, 5]. Anterior mediastinal mass is mainly related to T-lineage LBL or lymphoblastic leukaemia [3] and less commonly with B-cell LBL [6]. The prognosis of the adolescent-adult patients reported previously [1, 2] seems to be quite encouraging as compared to the index case. Probably the younger age is not a favourable factor. Another interesting fact could be the gender predisposition as all of the three reported cases are males. However, the numbers are too small for this to be conclusive. Since cytogenetic and molecular diagnostic data is not available, the cause of the poor outcome in this case cannot be explained. With a long history of 3–4 weeks this may also indicate the pitfalls of delayed diagnosis leading to life-threatening complications, advanced stage of disease and long-term poor prognosis.

## Conclusion

This is the first case of a paediatric B-cell ALL that presented with superior mediastinal syndrome secondary to an anterior mediastinal mass. This case concludes that B-cell ALL could also be a possible underlying diagnosis in paediatric anterior mediastinal masses. It also highlights the importance of early diagnosis before the development of life-threatening complications and advanced stage of disease at presentation.

## Conflicts of interest and funding

No conflicts of interest or funding to disclose.

## Figures and Tables

**Table 1.** Reported cases of B-cell ALL presenting with an anterior mediastinal mass.

Author/Year	Age/gender	Presenting complaints	Imaging	Blood counts/peripheral blood film	Diagnostic tests	Treatment and outcome
Pei *et al* [1]	20 years/male	Dyspnoea	Anterior mediastinal mass with massive pleural effusion	Normal, no blasts seen	Pleural fluid cytology showed lymphoma-like cells, identified as pre-B lymphoblasts on immunophenotyping. Immunophenotyping on bone marrow showed 42% precursor B lymphoblasts	Treated on modified GMALL 02/84 protocol. Disease-free for more than 7 years
Nupur *et al* [2]	16 years/male	Fever, dyspnoea, weight loss	Anterior mediastinal mass and right-sided pleural effusion. Retroperitoneal lymphadenopathy with deposits in both kidneys	Normal, no blasts seen	Bone marrow examination showed near-total replacement with small round blue cells (Burkitt-like morphology) Immunohistochemistry favoured Ewing Sarcoma but flow cytometric analysis showed Pre-B ALL. Extended immunostaining panel on bone marrow and lymph node biopsy confirmed CD45− FLI1+ MIC2+ B-ALL	BFM-90 protocol. Day 21 MRD showed no leukaemic cells

GMALL: German Multi enter ALL protocol; FLI1: Friend leukemia virus integration 1; MIC2: CD 99/ Cluster of differentiation 99 also known as MIC2; BFM-90: Berlin-Frankfurt-Munich 90 protocol
